# Macrophages and Autoantibodies in Demyelinating Diseases

**DOI:** 10.3390/cells10040844

**Published:** 2021-04-08

**Authors:** Haruki Koike, Masahisa Katsuno

**Affiliations:** Department of Neurology, Nagoya University Graduate School of Medicine, Nagoya 466-8550, Japan; ka2no@med.nagoya-u.ac.jp

**Keywords:** demyelination, electron microscopy, macrophage, paranode, pathogenesis, pathology, Schwann cell, the node of Ranvier, treatment

## Abstract

Myelin phagocytosis by macrophages has been an essential feature of demyelinating diseases in the central and peripheral nervous systems, including Guillain–Barré syndrome (GBS), chronic inflammatory demyelinating polyneuropathy (CIDP), and multiple sclerosis (MS). The discovery of autoantibodies, including anti-ganglioside GM1 antibodies in the axonal form of GBS, anti-neurofascin 155 and anti-contactin 1 antibodies in typical and distal forms of CIDP, and anti-aquaporin 4 antibodies in neuromyelitis optica, contributed to the understanding of the disease process in a subpopulation of patients conventionally diagnosed with demyelinating diseases. However, patients with these antibodies are now considered to have independent disease entities, including acute motor axonal neuropathy, nodopathy or paranodopathy, and neuromyelitis optica spectrum disorder, because primary lesions in these diseases are distinct from those in conventional demyelinating diseases. Therefore, the mechanisms underlying demyelination caused by macrophages remain unclear. Electron microscopy studies revealed that macrophages destroy myelin as if they are the principal players in the demyelination process. Recent studies suggest that macrophages seem to select specific sites of myelinated fibers, including the nodes of Ranvier, paranodes, and internodes, for the initiation of demyelination in individual cases, indicating that specific components localized to these sites play an important role in the behavior of macrophages that initiate myelin phagocytosis. Along with the search for autoantibodies, the ultrastructural characterization of myelin phagocytosis by macrophages is a crucial step in understanding the pathophysiology of demyelinating diseases and for the future development of targeted therapies.

## 1. Introduction

Macrophages play an important role, not only in normal immune system maintenance, but also in pathological conditions. Myelin phagocytosis by macrophages has been an essential feature of demyelinating diseases in the central and peripheral nervous systems, including Guillain–Barré syndrome (GBS), chronic inflammatory demyelinating polyneuropathy (CIDP), and multiple sclerosis (MS) [1,2,3,4,5]. Particularly, early ultrastructural studies, using biopsy specimens from patients with GBS and CIDP, have demonstrated the stripping of morphologically normal myelin lamellae by cytoplasmic processes of macrophages [1,6]. These macrophages caused researchers to assume that they are active players in the disease process, rather than mere scavengers of unnecessary materials. The discovery of autoantibodies directed against the constituents of the nervous system, by later researchers, contributed to the understanding of the disease process in a subpopulation of patients diagnosed with these diseases. For example, a concept that molecular mimicry between gangliosides located at the axolemma and the surface epitopes of exogenous pathogens induces the production of anti-ganglioside antibodies has been established in the axonal form of GBS [7]. Recent studies revealed that IgG4 autoantibodies against paranodal junction proteins found in a subpopulation of patients diagnosed with CIDP cause aberrant nerve conduction owing to paranodal dissection [8,9]. Regarding diseases of the central nervous system, anti-aquaporin 4 (AQP4) antibodies were found to be positive in patients with neuromyelitic optica [10]. However, lesions in the nervous system caused by these antibodies are distinct from those of conventional demyelination caused by macrophages [8,11,12]. Hence, the mechanisms underlying demyelination owing to myelin phagocytosis by macrophages remain unclear.

In this article, the mechanisms of demyelinating diseases are described by focusing on the role of macrophages and autoantibodies.

## 2. Role of Macrophages and Autoantibodies in Demyelinating Diseases

### 2.1. GBS

GBS is an acute polyneuropathy, which typically occurs following infection [13]. This disease was initially considered to be a demyelinating neuropathy, called acute inflammatory demyelinating polyneuropathy (AIDP), because a previous report demonstrated demyelinating lesions owing to myelin phagocytosis by macrophages [1]. Later studies revealed the presence of an axonal counterpart without the macrophage-associated demyelination as another form of GBS [11,14,15]. This axonal form of GBS is called acute motor axonal neuropathy (AMAN) or acute motor sensory axonal neuropathy depending on the absence or presence of sensory involvement [11]. The concept of molecular mimicry was established through studies on AMAN with anti-ganglioside GM1 antibodies that occurred after a *Campylobacter jejuni* infection [13]. The widely accepted theories are that the attachment of IgG autoantibodies to GM1, which is localized to the axolemma of motor fibers, and subsequent activation of complement cascades result in motor dysfunction in patients with AMAN [13,16,17]. Although the autopsy specimens revealed the presence of macrophages in the periaxonal space, macrophage-associated demyelinating lesions were not found in patients with AMAN [11].

Conversely, an association between specific autoantibodies with the occurrence of macrophage-associated demyelination in AIDP has not been clearly demonstrated. However, the presence of antecedent infections in patients with AIDP is similar to that in patients with AMAN, which indicates similar immunological processes [18]. An association between antibodies against moesin, which is expressed in the microvilli of Schwann cells at the nodes of Ranvier, and AIDP has been suggested after cytomegalovirus infection [19]. An increased occurrence of AIDP after the Zika virus infection has also been demonstrated [20]. Although an association between a specific anti-ganglioside antibody and Zika virus-related GBS has not been demonstrated [21], peptide sharing was suggested among proteins of the Zika virus, cytomegalovirus, and the human peripheral nervous system [22,23]. Recently, a conflicting discussion on the association between SARS-CoV2 infection (i.e., COVID-19) and AIDP has become a topic of research [24,25,26].

### 2.2. CIDP

CIDP has been a chronic counterpart of AIDP because similar macrophage-associated demyelination was reported as a pathological hallmark [2,4]. In contrast to AIDP, this disease is rarely accompanied by antecedent infections. CIDP was initially defined as neuropathy with a diffuse weakness of the limbs [2,27]. This classic form of CIDP was designated as “typical CIDP” in the criteria proposed by the European Federation of Neurological Societies and Peripheral Nerve Society (EFNS/PNS) and it is now frequently used in daily practice [28]. In addition to the typical CIDP, the EFNS/PNS criteria define five forms of “atypical CIDP”, which comprised multifocal acquired demyelinating sensory and motor (MADSAM), distal acquired demyelinating symmetric (DADS), pure sensory, pure motor, and focal subtypes [28]. Macrophage-associated demyelination was found not only in a typical CIDP but also in major atypical CIDP subtypes, including MADSAM, DADS, and pure sensory subtypes—although it is not found in all patients [29].

Recent studies demonstrated the presence of autoantibodies against paranodal junction components, including neurofascin 155 and contactin 1, in some patients diagnosed with typical CIDP and DADS [8,30,31,32,33,34]. In patients with these antibodies, aberrant nerve conduction is caused by the detachment of paranodal myelin terminal loops from the axolemma resulting from the deposition of autoantibodies to the paranodal junctions [4,8,35,36]. Unlike AMAN, the deposition of complements is not found at the paranodes because the immunoglobulin subclass of these antibodies is IgG4 [8]. Because classical demyelinating lesions associated with macrophages are not observed in patients with these antibodies, the concept of nodopathy or paranodopathy has recently been proposed for these patients [9,34]. However, patients with these antibodies constitute a minority in the total CIDP population [8,32,33]. The mechanisms underlying macrophage-associated demyelination remain to be elucidated.

### 2.3. MS and Related Diseases

MS is an inflammatory demyelinating disease of the central nervous system [3,37]. Based on the mode of progression, this disease is classified into four types: clinically isolated syndrome, relapsing remitting MS, primary progressive MS, and secondary progressive MS [38]. Although MS has traditionally been considered a disease mediated by T cells, particularly CD4-positive T cells reactive to myelin antigens [39], a recent study regarding the efficacy of rituximab, a chimeric monoclonal antibody to CD20, in patients with relapsing remitting MS suggested that B cells also play an important role in the pathogenesis of this disease [40]. In actual fact, the presence of oligoclonal IgG bands in the cerebrospinal fluid and deposition of IgG in active lesions have long been known as hallmarks of this disease [3]. Additionally, increasing evidence suggests that macrophages derived from circulating monocytes and resident macroglia play a pivotal role in the pathogenesis of MS [41,42]. Autopsy specimens from patients with MS revealed macrophages containing myelin debris in active lesions [43].

Whether neuromyelitis optica (also known as Devic’s disease), that preferentially affects the optic nerve and spinal cord, is a subtype of MS or an independent disease entity has been a controversy for a long time [44]. The discovery of disease-specific antibodies against AQP4 in the sera from patients with neuromyelitis optica resulted in significant progress regarding this concern [10]. Because AQP4 is expressed in astrocyte foot processes at the blood–brain barrier [45], pathological findings of AQP4 are characterized by perivascular lesions accompanied by deposition of IgG and complement [12]. Compared with demyelinating lesions found in conventional MS, the myelinated fibers are relatively preserved in these lesions [12]. Based on these findings, neuromyelitis optica is now regarded as a primary astrocytopathy and is distinct from MS. As anti-AQP4 antibodies are found to be associated with not only lesions in the optic nerves and spinal cord, but also those in other sites of the central nervous system, the concept of neuromyelitis optica spectrum disorder (NMOSD) has been proposed [46].

## 3. Morphology of Macrophages in Demyelination

**Figure 1:**
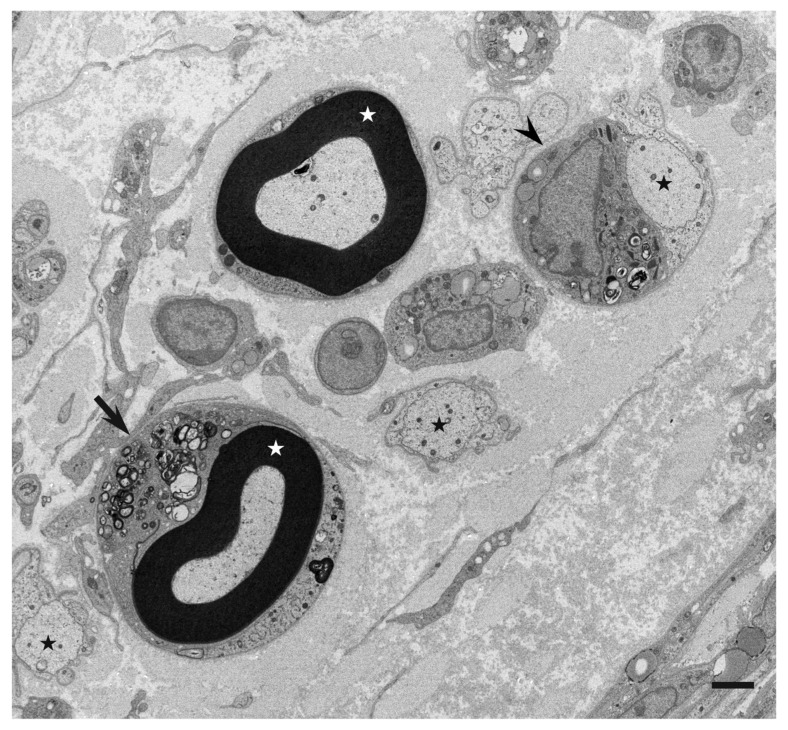
Representative electron microscopy photograph of demyelination caused by myelin phagocytosis by macrophages. A cross section of a sural nerve biopsy specimen obtained from a patient with AIDP. Various stages of demyelination are observed. The arrow indicates a myelinated fiber surrounded by the cytoplasm of macrophage containing myelin debris. Bold black circles indicated by white asterisks are myelin. The arrowhead indicates a macrophage that completed demyelination. Demyelinated axons are indicated by black asterisks. Uranyl acetate and lead citrate staining. Scale bar = 2 μm.

Ultrastructural studies using nerve biopsy specimens obtained from patients with the demyelinating form of GBS (i.e., AIDP) and those with CIDP have demonstrated putative chronological sequence in the progression of demyelination resulting from myelin phagocytosis by macrophages [4,5]. Generally, the morphology and behavior of macrophages participating in the demyelination process seem to be similar between AIDP and CIDP [4,5]. Various stages of demyelination may be seen in a single specimen (Figure 1), particularly in specimens from patients with CIDP [4].

**Figure 2:**
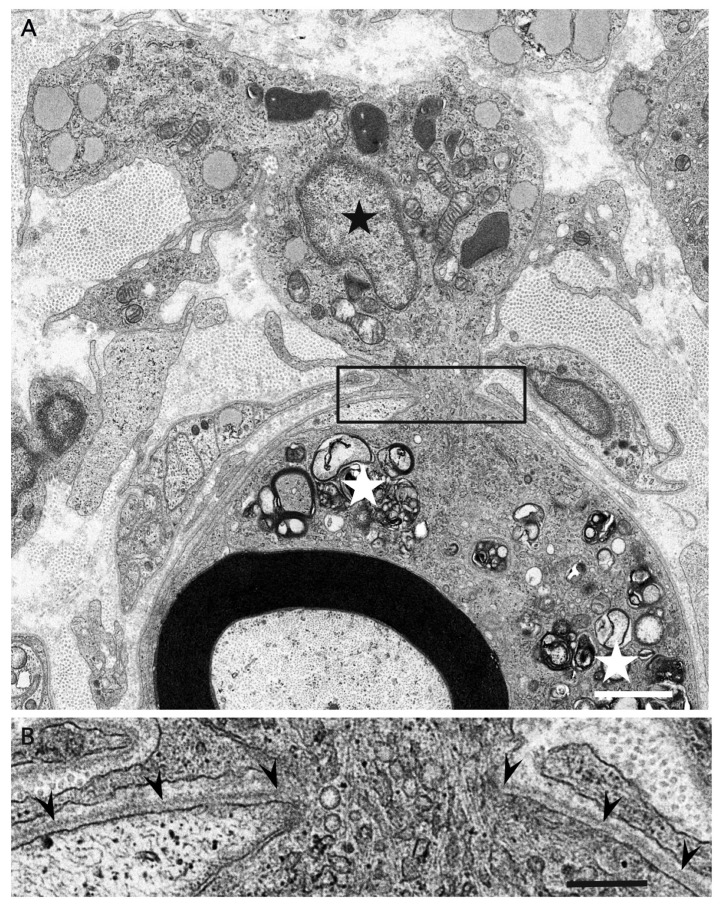
A macrophage invading the basement membrane tube surrounding the myelinated fiber. A cross section of a sural nerve biopsy specimen obtained from a patient with AIDP. A macrophage whose nucleus is indicated by a black asterisk is invading the basement membrane tube that normally surrounds myelinated fibers. Along with the invasion into the basement membrane tube, the cytoplasm of macrophages apposed to myelin initiates degradation of myelin (white asterisks). Note that the cytoplasm of this macrophage located outside the basement membrane tube does not contain myelin debris. A high-powered view of the region in the box in (**A**) is shown in (**B**). Basement membranes surrounding myelinated fibers are indicated by arrowheads. Uranyl acetate and lead citrate staining. Scale bars = 2 μm (**A**) and 0.5 μm (**B**).

**Figure 3:**
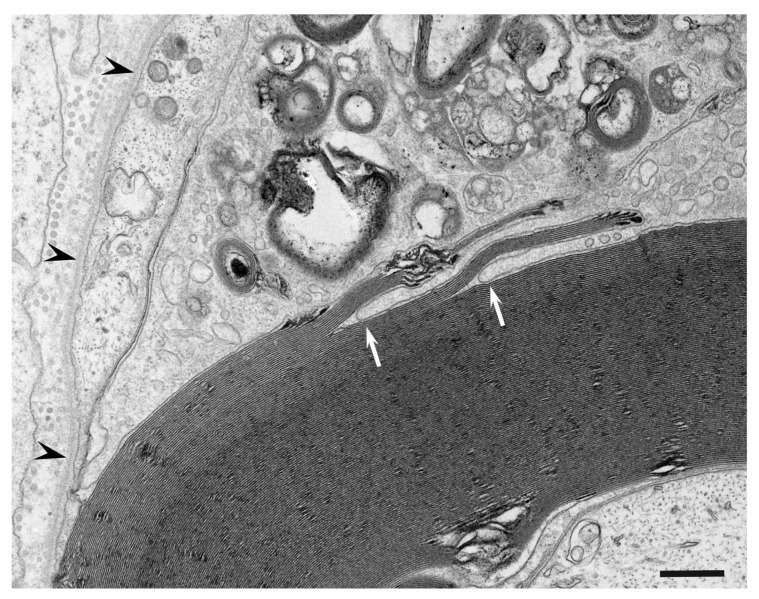
Stripping of the myelin lamellae by a cytoplasmic process of the macrophage. A cross section of a sural nerve biopsy specimen obtained from a patient with AIDP. Cytoplasmic processes of the macrophage indicated by arrows peel off the myelin layers. A basement membrane surrounding the myelinated fibers is indicated by arrowheads. Uranyl acetate and lead citrate staining. Scale bar = 0.5 μm.

Although resident macrophages are present in the peripheral nervous system, blood-derived macrophages that enter the endoneurium under the guidance of adhesion molecules, such as ICAM-1, VCAM-1, and ELAM-1, can also participate in the demyelination process [47,48,49,50]. Morphological distinction between these macrophages has not been clearly established despite their functional differences [51]. Macrophages approach the myelinated fibers and extend their cytoplasmic processes to enter the basement membrane tube surrounding the myelinated fibers (Figure 2) [4]. A recent review by Park et al. suggested that the entry sites of macrophages are limited to where myelin lamellae are partially degenerated or separated from Schwann cell cytoplasm [52]. Along with invading the basement membrane tube, the cytoplasm of macrophages apposed to myelin initiates degradation of the myelin lamellae. Macrophages seem to peel off layers of myelin using their cytoplasmic processes (Figure 3) [4,5]. Unraveling and disruption of the myelin lamellae apposed to the cytoplasm of macrophages are also frequently observed [4,5,53].

**Figure 4:**
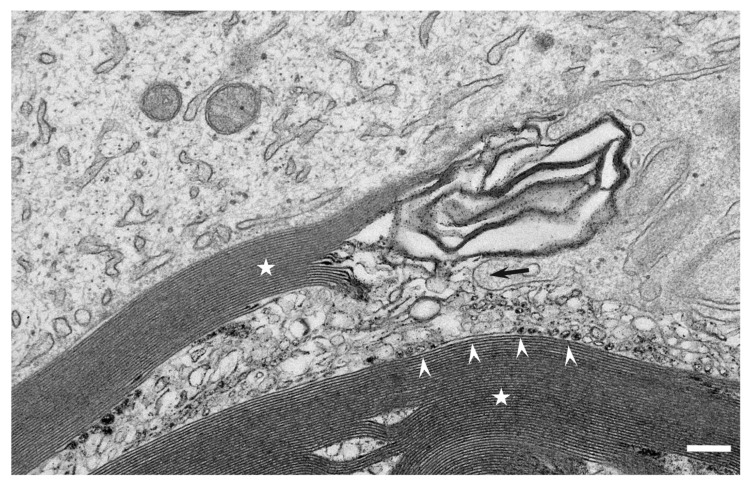
Vesicular dissolution of myelin. A cross section of a sural nerve biopsy specimen obtained from a patient with AIDP. Vesicular dissolution of the myelin is seen in a space between the myelin lamellae indicated by asterisks. Vesicles seem to be formed by the separation of the major dense lines (arrowheads). A process of macrophage indicated by an arrow seems to be invading a gap created by the dissolution of myelin lamellae. Uranyl acetate and lead citrate staining. Scale bar = 0.2 μm.

**Figure 5:**
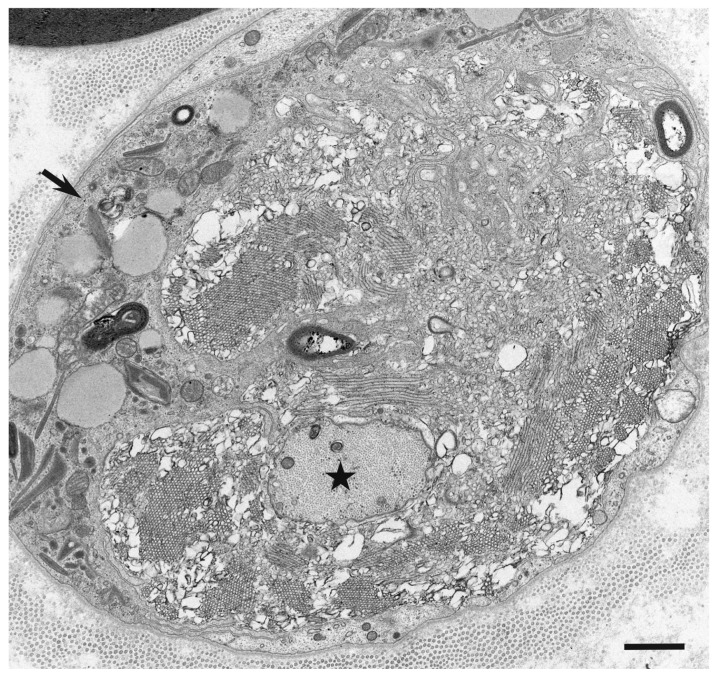
A demyelinated axon. A cross section of a sural nerve biopsy specimen obtained from a patient with AIDP. A demyelinated axon indicated by an asterisk is surrounded by a space filled with vesicular dissolution of the myelin. The cytoplasm of a macrophage indicated by an arrow is also within the basement membrane tube. Uranyl acetate and lead citrate staining. Scale bar = 1 μm.

Vesicular dissolution of the myelin has been reported as another important lesion associated with demyelinating diseases, including AIDP and MS (Figure 4) [1,54,55,56,57,58]. Most studies describing this finding used autopsy specimens [54,55,56,57,58]. One of these studies used specimens from patients with AIDP, which demonstrated that the vesicular dissolution occurred where complements were deposited but macrophages were absent [56]. Therefore, this finding might be an early morphological change occurring before a macrophage invasion into the basement membrane tube of the myelinated fibers. However, similar findings also seem to be closely associated with macrophages invading the basement membrane tube (Figure 5) [5,59]. Therefore, hydrolases released from macrophages may be involved in myelin lesions, including unraveling, disruption, and vesicular dissolution.

**Figure 6:**
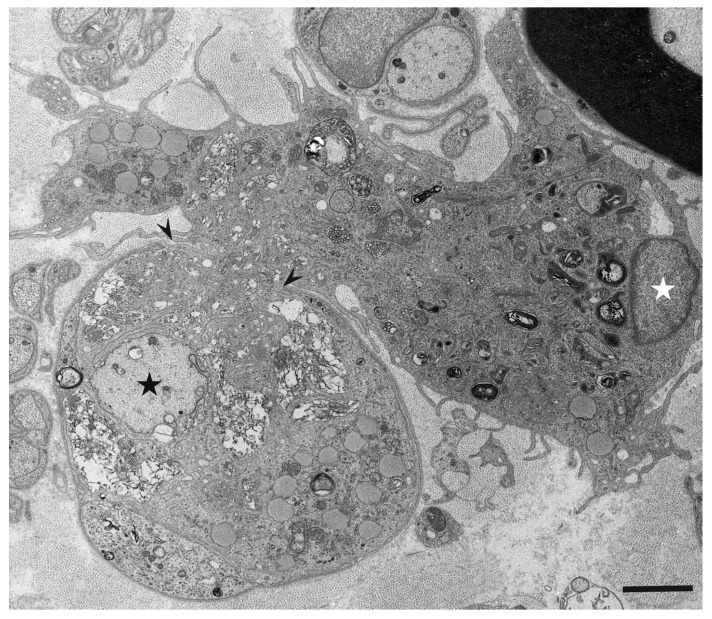
A macrophage escaping from the basement membrane tube surrounding the myelinated fiber. A cross section of a sural nerve biopsy specimen obtained from a patient with AIDP. The sites at which the basement membrane was disrupted are indicated by arrowheads. The nucleus of this macrophage is located outside of the basement membrane tube. Note that an axon located within the basement membrane tube is completely demyelinated. A demyelinated axon and a macrophage nucleus are indicated by a black asterisk and a white asterisk, respectively. Uranyl acetate and lead citrate staining. Scale bar = 2 μm.

Despite these findings resulting in the breakdown of compacted myelin lamellae, the uncompacted Schwann cell cytoplasm located outside the myelin lamellae remains intact [4]. Once the myelin breakdown is completed, macrophages containing myelin debris penetrate the basement membrane again to escape to the outer space (Figure 6) [4,5].

A similar demyelination process has also been reported in studies of experimental allergic neuritis, which is an experimental model of GBS or CIDP [60,61], and experimental allergic encephalomyelitis: a model of MS [62,63,64].

## 4. What Attracts Macrophages to Myelin?

Unlike that on anti-ganglioside GM1 antibodies in AMAN, anti-neurofascin 155 antibodies in nodopathy or paranodopathy, and anti-AQP4 antibodies in NMOSD, knowledge of the relationship between specific autoantibodies and demyelination caused by macrophages is still limited. An association between antibodies against moesin, which is expressed at the microvilli of the Schwann cells at the nodes of Ranvier, and AIDP following cytomegalovirus infection has been suggested [19]. Gliomedin, galactocerebroside, and ganglioside LM1 have also been suggested as target antigens in AIDP [65,66,67,68]. Although recent emerging infectious diseases, including Zika virus infection and COVID-19, are reported to be associated with AIDP rather than AMAN [20,26], causative autoantibodies associated with these viruses have not been detected to date. Regarding CIDP, sural nerve biopsy specimens from a patient with antibodies to LM1 ganglioside, which is abundant in myelin, revealed complement deposition on myelin and demyelination by macrophages [69]. However, patients with anti-LM1 antibodies constitute only a minority of the total CIDP population [70]. Recent studies revealed that antibodies to myelin oligodendrocyte glycoprotein (MOG), expressed in the outermost layer of the myelin sheath, were found in some of the patients with NMOSD negative for anti-AQP4 antibodies [71]. The location of the target antigen suggests myelin damage, which is distinct from astrocyte damage in NMOSD positive for anti-AQP4 antibodies [12]. However, patients with conventional MS are typically negative for anti-MOG antibodies [72].

Based on the abovementioned results, the mechanism underlying demyelination, resulting from myelin phagocytosis by macrophages, remains an enigma from the viewpoint of autoantibodies. A recent electron microscopy study using longitudinal sections of biopsy specimens from patients with AIDP suggested that macrophages seemed to select specific sites of myelinated fibers, including the nodes of Ranvier, paranodes, and internodes, for the initiation of demyelination in individual cases [5]. The sites of complement deposition corresponded to the distribution of macrophages in that study, suggesting the presence of undiscovered autoantibodies directed against the components of myelinated fibers in AIDP [5]. The efficacy of eculizumab, a humanized monoclonal antibody to complement component 5, for not only AMAN, but also AIDP, supports this view [73]. Similar selectivity of the sites at which macrophages initiate myelin phagocytosis was also reported in CIDP [4]. However, the pathogenesis of CIDP might be more complex than that of AIDP, considering the heterogeneity of its clinical features and its response to immunotherapies [9]. The mechanisms of demyelination in MS are also considered complex, involving both innate and adoptive (i.e., humoral and cellular) immunities [37,41]. Additionally, it has been gradually established that macrophages not only contribute to the initiation and development of demyelination by boosting inflammatory events, but they also play a protective role by suppressing inflammation, eliminating debris, and promoting repair [74]. Particularly, immunoregulatory M2 macrophages are considered to be predominant during the recovery and repair process [75]. Further studies focusing on the mechanisms leading to myelin phagocytosis by macrophages are required to elucidate the pathogenesis of demyelinating diseases.

## 5. Summary and Conclusions

Myelin phagocytosis by macrophages has traditionally been considered an essential feature of demyelinating diseases of the central and peripheral nervous systems, including GBS, CIDP, and MS [1,2,3,4,5]. The discovery of autoantibodies directed against the constituents of the nervous system contributed to the understanding of the disease process in a subpopulation of patients conventionally diagnosed with these diseases. For example, a concept that molecular mimicry between gangliosides located at the axolemma and the surface epitopes of exogenous pathogens induces the production of anti-ganglioside antibodies has been established in the axonal form of GBS, called AMAN [7,13]. In patients with AMAN, the attachment of autoantibodies to ganglioside GM1 localized to the axolemma of motor fibers and subsequent activation of complement cascades result in the conduction failure of the motor nerve fibers [13,16,17]. In a subpopulation of patients with CIDP, autoantibodies against paranodal junction components, including neurofascin 155 and contactin 1, cause aberrant nerve conduction, owing to the detachment of paranodal myelin terminal loops from the axolemma [8]. As demyelinating lesions associated with macrophages are not found in patients with these antibodies, the concept of nodopathy or paranodopathy has recently been proposed [9,34]. Moreover, NMOSD associated with anti-AQP4 antibodies is now considered a disease entity distinct from MS because astrocytes, rather than oligodendrocytes, are primarily affected [12].

Despite the discovery of these antibodies, the mechanisms underlying demyelination owing to myelin phagocytosis by macrophages remain unclear. Ultrastructural studies revealed that macrophages strip morphologically intact myelin lamellae by their cytoplasmic processes as if they are principal players in the demyelination process [4,5]. Unraveling, disruption, and vesicular dissolution of the myelin lamellae are also frequently observed where the cytoplasm of macrophages is present [5], as if hydrolases released from macrophages may also be involved. Recent electron microscopy studies using longitudinal sections of biopsy specimens from patients with AIDP and those with CIDP suggested that macrophages seemed to select specific sites of myelinated fibers, including the nodes of Ranvier, paranodes, and internodes, for the initiation of demyelination in individual cases [4,5]. Hence, specific components localized to these sites may play an important role in the behavior of macrophages that initiate myelin phagocytosis. Along with the search for autoantibodies, the ultrastructural characterization of myelin phagocytosis by macrophages is a crucial step in understanding the pathophysiology of demyelinating diseases and for the future development of targeted therapies.
